# Phylogeny of Thaumastodermatidae (Gastrotricha: Macrodasyida) Inferred from Nuclear and Mitochondrial Sequence Data

**DOI:** 10.1371/journal.pone.0017892

**Published:** 2011-03-24

**Authors:** M. Antonio Todaro, Tobias Kånneby, Matteo Dal Zotto, Ulf Jondelius

**Affiliations:** 1 Department of Biology, University of Modena and Reggio Emilia, Modena, Italy; 2 Department of Invertebrate Zoology, Swedish Museum of Natural History, Stockholm, Sweden; British Columbia Centre for Excellence in HIV/AIDS, Canada

## Abstract

**Background:**

Phylogenetic relationships within Gastrotricha are poorly known. Attempts to shed light on this subject using morphological traits have led to hypotheses lacking satisfactory statistical support; it seemed therefore that a different approach was needed.

**Methodology/Principal Findings:**

In this paper we attempt to elucidate the relationships within the taxonomically vast family Thaumastodermatidae (Macrodasyida) using molecular sequence data. The study includes representatives of all the extant genera of the family and for the first time uses a multi-gene approach to infer evolutionary liaisons within Gastrotricha. The final data set comprises sequences of three genes (18S, 28S rDNA and COI mtDNA) from 41 species, including 29 thaumastodermatids, 11 non-thaumastodermatid macrodasyidans and a single chaetonotidan. Molecular data was analyzed as a combined set of 3 genes and as individual genes, using Bayesian and maximum likelihood approaches. Two different outgroups were used: *Xenotrichula intermedia* (Chaetonotida) and members of the putative basal *Dactylopodola* (Macrodasyida). Thaumastodermatidae and all other sampled macrodasyidan families were found monophyletic except for Cephalodasyidae. Within Thaumastodermatidae Diplodasyinae and Thaumastodermatinae are monophyletic and so are most genera. *Oregodasys* turns out to be the most basal group within Thaumastodermatinae in analyses of the concatenated data set as well as in analyses of the nuclear genes. *Thaumastoderma* appears as the sister taxon to the remaining species. Surprisingly, *Tetranchyroderma* is non-monophyletic in our analyses as one group of species clusters with *Ptychostomella* while another appears as the sister group of *Pseudostomella*.

**Conclusions/Significance:**

Results in general agree with the current classification; however, a revision of the more derived thaumastodermatid taxa seems necessary. We also found that the ostensible COI sequences from several species do not conform to the general invertebrate or any other published mitochondrial genetic code; they may be mitochondrially derived nuclear genes (numts), or one or more modifications of the mitochondrial genetic code within Gastrotricha.

## Introduction

The approximately 760 described species of Gastrotricha are small aquatic metazoans. The group is cosmopolitan and a common component of the meiofauna. Gastrotrichs have been classified as Rotifera [1], Ciliata [2], Platyhelminthes [3] or closely related to Nematoda [4]. Hyman [5] and Ruppert [6] regarded the group as a class within Aschelminthes. Today Gastrotricha is considered a phylum [7], which based on molecular data, most likely has a basal position in the Platyzoa within Lophotrochozoa [e.g. 8]–[11]. Recent phylogenomic studies by Dunn *et al.* [12] and Hejnol *et al.* [13] also place Gastrotricha within Platyzoa. A competing hypothesis regards the group as affiliated to Ecdysozoa based on morphology [e.g. 14], [15] and morphology together with molecular data [16].

Gastrotricha currently contains two orders, Chaetonotida and Macrodasyida, based on morphological data [17]. Members of Chaetonotida are tenpin-shaped and contain both marine and freshwater species. Macrodasyidans are worm-shaped and almost exclusively marine. The two orders were both considered monophyletic on morphological grounds by Hochberg & Litvaitis [18]. The recent comprehensive morphological study by Kieneke *et al.* [19] hypothesizes Macrodasyida, with the exclusion of the enigmatic freshwater *Redudasys* and *Marinellina*, to be monophyletic. However, molecular and morphological analyses are not fully congruent since Macrodasyida and Chaetonotida are often resolved as non-monophyletic groups [e.g. 11], [16], [20], [21]. Manylov *et al.* [22] investigated the differences between the two gastrotrich orders based on 18S rDNA and suggested a monophyletic Macrodasyida and a monophyletic Paucitubulatina (a suborder together with Multitubulatina within Chaetonotida). However these groups clustered with different bilaterian taxa and Gastrotricha was considered a polyphyletic taxon.

The evolutionary relationships among and within the lower ranking taxa (e. g. families and genera) of Gastrotricha, including the ones that from a morphological point of view are quite well investigated, remain virtually unknown. A good example in this regard is the marine Thaumastodermatidae, the most speciose family within Macrodasyida, with more than 130 species [7]. Thaumastodermatid gastrotrichs are geographically widespread and live interstitially in coarse shelly and medium to fine grained subtidal or intertidal sand. These marine gastrotrichs can easily be identified because of their relatively large mouth, two posterior adhesive pedicles and especially their extraordinary cuticle, which forms spines, sculpted plates, bowl-shaped scales or multi-spined scales. Other, less-immediate, characteristics include the pharynx with reduced radial musculature and small pharyngeal pores, the lack of somatic circular muscles posterior to the head region, an internal connection of the vasa deferentia or vas deferens to the caudal organ and multiciliated epidermal cells [23]. Within the family eight genera are currently recognized and distributed into two subfamilies: *Acanthodasys* and *Diplodasys* (Diplodasyinae) vs *Hemidasys*, *Oregodasys( = Platydasys)*, *Pseudostomella*, *Ptychostomella*, *Tetranchyroderma* and *Thaumastoderma* (Thaumastodermatinae). *Hemidasys* was described by Claparède [24] is now considered extinct [7].

Members of Diplodasyinae may be distinguished because they have, among other characters, paired testes and frontal and caudal organ that are anatomically and functionally disjunct i.e. the frontal organ ( = frontal sac) is located at mid-body, in front of the largest egg, whereas the caudal organ is located in the posterior trunk region. In contrast, species of Thaumastodermatinae possess a single testis on the right side of the body and the frontal- and caudal organs are anatomically (and functionally?) close to (attached) each other, in the posterior trunk region [23].

Since gastrotrichs rather recently gained attention in molecular studies, most phylogenetic hypotheses dealing with the group are based on morphology only. For instance, Hochberg & Litvaitis [18] using a parsimony analysis of 81 morphological characters found Thaumastodermatidae to be monophyletic, suggesting two autapomorphies for the family: (i) sperm ducts that internally connect to the caudal organ, (ii) a wide bulging buccal cavity. Moreover, the two subfamilies introduced by Ruppert [23] were found monophyletic. Also Kieneke *et al.* [19] using a parsimony analysis of 135 morphological characters found a monophyletic Thaumastodermatidae, but the monophyly of the two subfamilies was not recovered in their phylogenetic tree. It should be highlighted however that the topology obtained by Kieneke *et al.* [19] was plagued by low bootstrap support at most nodes.

The monophyly of Thaumastodermatidae and the family's internal relationships have never been purposely tested with a molecular approach, and in general, taxon sampling with regard to this family has been very poor in the previous gastrotrich molecular studies i.e. 1–2 species involved. One possible exception is the study by Todaro *et al.* [11], where six of the 43 gastrotrich taxa examined were thaumastodermatid species. In that study, the analysis of near complete and partial 18S rDNA yielded Thaumastodermatidae and the two subfamilies as monophyletic [11]. These preliminary encouraging results seemed to call for a widening of the molecular study in order to get a complete picture about relationships within the family. Consequently, we arranged to obtain specimens belonging to species of all the extant thaumastodermatid genera, and in an attempt to provide robustness to the outcomes, we planned the phylogenetic analyses to be based on comparison of multi-gene sequences.

## Materials and Methods

### Selection of taxa

**Figure 1:**
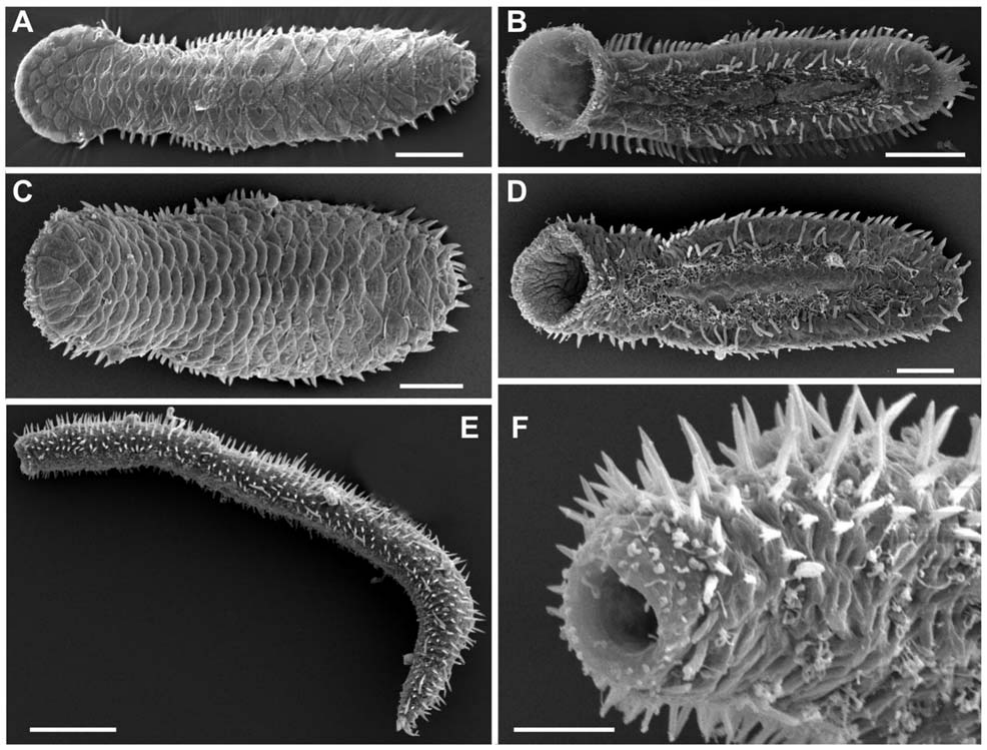
Gastrotricha, Thaumastodermatidae, Diplodasyinae. SEM photomicrographs showing the general body shape and aspects of the cuticular covering of representatives of the genera *Diplodasys* and *Acanthodasys*. A, B, *Diplodasys ankeli*, dorsal and ventral view respectively; C, D, *Diplodasys* sp. from Kuwait, dorsal and ventral view respectively; E, F, *Acanthodasys aculeataus*, habitus and close-up of the anterior end. Scale bars A, B, E = 50 µm, C, D = 20 µm, F = 10 µm.

To estimate the interrelationships within the family Thaumastodermatidae, complete 18S rDNA, partial 28S rDNA and COI mtDNA genes were sequenced from 29 single specimens, representative of all eight extant genera and including at least two species per genus (24 spp in total), with the exception of *Pseudostomella* for which only sequences of a single species were obtained (Figs. 1–3). In an attempt to determine possible intrageneric relationships, we decided to include several species of *Tetranchyroderma* (the far most speciose genus in the family), which may form 2–3 groups based on morphological traits such as type of cuticular covering, the presence and/or shape of cephalic sensorial organs etc., all characteristics that are widely used in dichotomous keys for species identification [e.g. 25].

**Figure 2 pone-0017892-g002:**
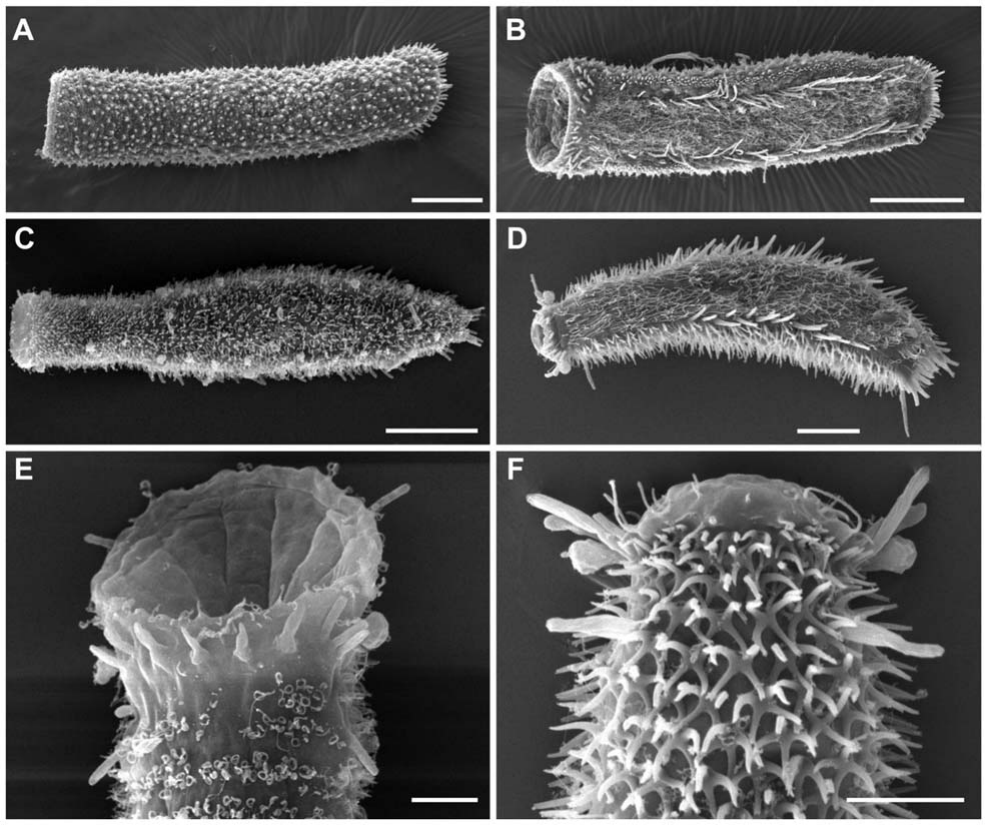
Gastrotricha, Thaumastodermatidae, Thaumastodermatinae. SEM photomicrographs showing the general body shape and aspects of the cuticular covering of representatives of the genera *Oregodasys*, *Tetranchyroderma* and *Thaumastoderma*. A, B, *Oregodasys ocellatus* dorsal and ventral view respectively; C, E *Tetranchyroderma* cf. *antennatum*, *C*, habitus dorsal view, E, close-up of the anterior end in a ventral view showing the ample mouth, adhesive tubes of the anterior series and cephalic sensory organ; D, F *Thaumastoderma ramuliferum*, D, habitus in ventral view; F, close-up of the anterior end in a dorsal view showing the cuticular armature made up of tetrancres, cephalic sensory organs and the cirrata tubes. Scale bars A–C = 50 µm, D–F = 20 µm.

**Figure 3:**
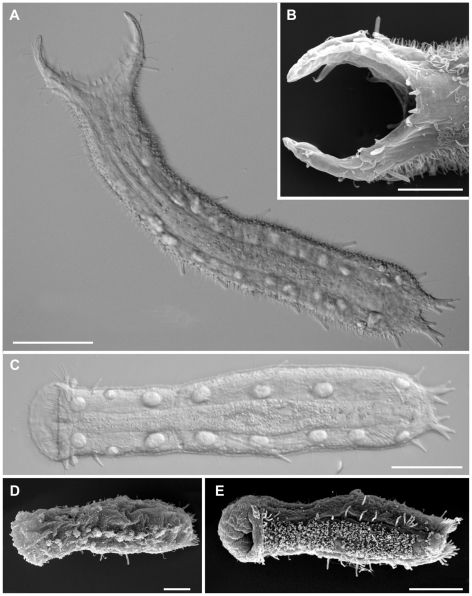
Gastrotricha, Thaumastodermatidae, Thaumastodermatinae. DIC (A, C) and SEM (B,D,E) photomicrographs showing the general body shape and aspects of the cuticular covering of representatives of the genera *Pseudostomella* and *Ptychostomella*. A, B, *Pseudostomella etrusca*, A, habitus, B, close-up of the anterior end showing the impressive oral palps; C, *Ptychostomella* sp 1, habitus; D, *Ptychostomella mediterranea*, habitus, dorsal view; E, *Ptychostomella* sp., habitus ventral view. The latter two species were not involved in the molecular study. Scale bars A, C = 50 µm, B, D, E = 20 µm.

The identity of the sister group of Thaumastodermatidae is not known. Based on similarity of some morphological traits, Ruppert [23] indicated *Lepidodasys* as the potential sister taxon of Thaumastodermatidae; this suggestion found some support by the cladistic analysis of Hochberg & Litvaitis [18]. However, Ruppert's hypothesis has not been substantiated by past molecular analyses, including that of Todaro *et al.* [11], which found the family in a sister group relationship with a cluster of chaetonotidans, although with low statistical support. In order to get some insights in this regard, we sampled representatives of five additional macrodasyidan families for a total of 19 specimens belonging to 16 species in seven genera.

Finally, a representative of the order Chaetonotida, *Xenotrichula intermedia* (Xenotrichulidae), was chosen as the out-group in the analyses. On morphological grounds, xenotrichulid gastrotrichs possess characteristics that are perceived to be plesiomorphic (e.g. solely marine, hermaphroditic sexual apparatus, functional spermatozoa, etc.) hence they are empirically considered among the basal taxa within Chaetonotida. Moreover, they are readily available in contrast with members of two other possible basal chaetonotidans families i.e., Neodasyidae and Muselliferidae, which are infrequent or rare [e.g. 26], [27]. To look for congruence, in some analyses members of the genus *Dactylopodola* (Dactylopodolidae) were used as outgroups since these gastrotrichs are thought to possess the ancestral macrodasyidan features and the family has always resulted to be a putative primitive lineage within the Macrodasyida in analyses based on morphology [e.g. 18], [28]–[31].

All of the specimens used in this study were found during a number of faunistic surveys headed by the senior author; no special permission/permits were needed to collect the animals under study. Soon after sampling, gastrotrichs were extracted from the sandy substrata using a 7% MgCl_2_ solution [17], fixed in 95% Ethanol and stored at −20°C until further treatment. Full list of specimens, together with sampling location as well as geographic coordinates and GenBank accession numbers are presented in Tables 1,2.

**Table 1:** Thaumastodermatidae taxa used in this study.

Taxon	Sampling location	Coordinates	GenBank accession number		
			18S	28S	COI
**Diplodasyinae**					
*Acanthodasys* sp. A	Capraia, Italy	43°00′53″N 09°49′24″E	JF357638	JF357686	NA
*Acanthodasys aculeatus*	Capraia, Italy	43°00′53″N 09°49′24″E	JF357639	JF357687	NA
*Diplodasys* sp.	Kuwait	28°32.59″N; 48°25′18″E	JF357641	JF357689	JF432032
*Diplodasys ankeli*	Meloria, Italy	43°33′11″N 10°13′20″E	JF357624	NA	NA
*Diplodasys ankeli*	Bohuslän, Sweden	See [59]	JF357667	NA	JF432049
*Diplodasys meloriae*	Meloria, Italy	43°33′11″N 10°13′20″E	JF357632	JF357688	NA
*Diplodasys meloriae*	Meloria, Italy	43°33′11″N 10°13′20″E	JF357640	JF357680	JF432031
**Thaumastodermatinae**					
*Oregodasys ocellatus*	Meloria, Italy	43°33′11″N; 10°13′20″E	JF357642	JF357690	NA
*Oregodasys ruber*	Meloria, Italy	43°33′11″N; 10°13′20″E	JF357625	JF357673	JF432020
*Oregodasys tentaculatus*	Meloria, Italy	43°33′11″N; 10°13′20″E	JF357626	JF357674	JF432021
*Pseudostomella etrusca*	Albinia, Italy	42°29′29″N; 11°11′28″E	JF357633	JF357681	JF432026
*Ptychostomella* sp. 1	Ilha Bela, Brazil	23°50′30″S; 45°24′14″W	JF357643	JF357691	JF432033
*Ptychostomella tyrrhenica*	Albinia, Italy	42°29′29″N; 11°11′28″E	JF357634	JF357682	JF432027
*Tetranchyroderma papii*	Sardegna, Italy	40°35′02″N; 08°15′37″E	JF357637	JF357685	JF432030
*Tetran.* cf. *antennatum*	Kuwait	28°32.59″N; 48°25′18″E	JF357645	JF357693	NA
*Tetran. cirrophorum*	Capraia, Italy	43°00′53″N; 09°49′24″E	JF357635	JF357683	JF432028
*Tetran. esarabdophorum*	Mahdia, Tunisia	35°30′57″N; 11°03′00″E	JF357627	JF357675	JF432022
*Tetran. hirtum*	Capraia, Italy	43°00′53″N; 09°49′24″E	JF357628	JF357676	JF432023
*Tetran. pachysomum*	Meloria, Italy	43°33′11″N; 10°13′20″E	JF357636	JF357684	JF432029
*Tetran. quadritentaculatum*	Albinia, Italy	42°29′22″N; 11°11′27″E	JF357629	JF357677	NA
*Tetran. quadritentaculatum*	Punta Ala, Italy	42°48′42″N; 10°44′46″E	JF357647	JF357695	JF432024
*Tetran. thysanophorum*	Albinia, Italy	42°29′22″N; 11°11′27″E	JF357630	JF357678	JF432025
*Tetran. thysanophorum*	Fautea, Corsica	41°42′40″N; 09°24′17″E	JF357646	JF357694	JF432034
*Tetranchyroderma* sp. 1	Bohuslän, Sweden	See [59]	JF357672	NA	NA
*Tetranchyroderma* sp. 3	Ilha Bela, Brazil	23°50′30″S; 45°24′14″W	JF357648	JF357696	JF432035
*Tetranchyroderma* sp. 4	Ilha Bela, Brazil	23°50′30″S; 45°24′14″W	JF357644	JF357692	NA
*Thaumastoderma moebjergi*	Bohuslän, Sweden	See [59]	JF357671	JF357713	NA
*Thaumast. ramuliferum*	Meloria, Italy	43°33′11″N; 10°13′20″E	JF357631	JF357679	NA
*Thaumast. ramuliferum*	Punta Ala, Italy	42°48′42″N; 10°44′46″E	JF357649	JF357697	NA

Sampling locations together with their respective coordinates are given as well as GenBank accession number. NA, Not available.

**Table 2:** Non-Thaumastodermatidae taxa used in this study.

Taxon	Sampling location	Coordinates	GenBank accession number		
			18S	28S	COI
**Cephalodasyidae**					
*Megadasys* sp.	Grotta del Ciolo, Italy	39°50′38″N; 18°23′09″E	JF357655	JF357703	JF432040
*Megadasys* sp. 1	Porto Cesareo, Italy	40°15′33″N; 17°53′53″E	JF357656	JF357704	JF432041
*Mesodasys laticaudatus*	Albinia, Italy	42°29′29″N; 11°11′28″E	JF357657	JF357705	JF432042
*Mesodasys laticaudatus*	Bohuslän, Sweden	See [59]	JF357668	NA	JF432050
*Mesodasys littoralis*	Bou Ficha, Tunisia	36°16′50″N; 10°29′41″E	JF357658	JF357706	JF432043
**Dactylopodolidae**					
*Dactylopodola* cf. *baltica*	Kuwait	29°20′53″N; 48°06′02″E	JF357650	JF357698	NA
*Dactylopodola mesotyphle*	Punta Ala, Italy	42°48′42″N; 10°44′46″E	JF357651	JF357699	JF432036
*Dactylopodola typhle*	Bou Ficha, Tunisia	36°16′50″N; 10°29′41″E	JF357652	JF357700	JF432037
*Dactylopodola typhle*	Torre Civette, Italy	42°50′42″N; 10°46′31″E	JF357653	JF357701	JF432038
**Lepidodasyidae**					
*Lepidodasys unicarenatus*	Pianosa, Italy	42°37′04″N 10°05′21″E	JF357665	NA	JF432048
**Macrodasyidae**					
*Macrodasys* sp. 1	Torre Civette, Italy	42°50′42″N 10°46′31″E	JF357654	JF357702	JF432039
*Macrodasys* sp. 2	Bohuslän, Sweden	See [59]	JF357670	JF357714	JF432052
**Turbanellidae**					
*Paraturbanella dohrni*	Punta Ala, Italy	42°48′42″N; 10°44′46″E	JF357659	JF357707	NA
*Paraturbanella pallida*	Capraia, Italy	43°00′53″N; 09°49′24″E	JF357660	JF357708	JF432044
*Paraturbanella teissieri*	Punta Ala, Italy	42°48′42″N; 10°44′46″E	JF357661	JF357709	NA
*Turbanella bocqueti*	Tramore, Ireland	52°09′24″N; 07°08′12″W	JF357662	JF357710	JF432045
*Turbanella cornuta*	Chioggia, Italy	45°12′57″N; 12°17′57″E	JF357663	JF357711	JF432046
*Turbanella cornuta*	Åhus, Sweden	55°54′22″N; 14°17′41″E	JF357666	NA	NA
*Turbanella lutheri*	Torö, Sweden	58°48′30″N; 17°48′20″E	JF357669	NA	JF432051
**Xenotrichulidae** *					
*Xenotrichula intermedia*	Mahdia, Tunisia	35°30′57″N; 11°03′00″E	JF357664	JF357712	JF432047

*order Chaetonotida.

Sampling locations together with their respective coordinates are given as well as GenBank accession number. NA, Not available.

### DNA extraction and amplification

DNA was extracted from single whole specimens using the QIAamp DNA mini kit (QIAGEN) with columns from the QIAamp DNA micro kit (QIAGEN) according to the manufacturer's instructions. The extraction yielded two extracts of 20 and 40 µl respectively for each specimen. DNA was amplified using the 0.2 ml PuReTaq Ready-To-Go PCR beads (GE Healthcare). For ribosomal 18S and 28S rDNA ∼1700 bp and ∼2500 bp were amplified respectively and for mitochondrial COI ∼660 bp. For amplification 0.5 µl of each primer, 2 µl of DNA and 22 µl of purified water were assembled in the RTG-PCR tubes yielding a final volume of 25 µl. Primer sequences and PCR-programs are presented in Table 3. Polymerase chain reactions were made in a Gene Amp PCR System 9700 (Applied Biosystems). For some COI sequences a reamplification was necessary to get a sufficient amount of DNA. PCR products were checked on a 0.8% ethidium-bromide gel.

**Table 3:** Primers used in this study and their respective direction, primer sequence as well as usage.

Primers & Regime	Direction	Primer sequence (5′-3′)	Usage	Reference
**18S/SSU Primers**				
S30	Forward	GCTTGTCTCAAAGATTAAGCC	PCR/Sequencing	[60]
5FK	Reverse	TTCTTGGCAAATGCTTTCGC	PCR/Sequencing	[60]
PCR regime S30/5FK	95°C at 4 min, 40×(94°C at 30 s, 52°C at 30 s, 72°C at 30 s), 75°C at 10 min			
4FB	Forward	CCAGCAGCCGCGGTAATTCCAG	PCR/Sequencing	[60]
1806R	Reverse	CCTTGTTACGACTTTTACTTCCTC	PCR/Sequencing	[60]
PCR regime 4FB/1806R	95°C at 4 min, 2×(94°C at 30 s, 60-52°C at 30 s, (2°C touch down) 72°C at 30 s), 30×(94°C at 30 s, 50°C at 30 s, 72°C at 30 s), 72°C at 10 min			
5F	Forward	GCGAAAGCATTTGCCAAGAA	Sequencing	[60]
7F	Forward	GCAATAACAGGTCTGTGATGC	Sequencing	[60]
7FK	Reverse	GCATCACAGACCTGTTATTGC	Sequencing	[60]
**28S/LSU Primers**				
U178	Forward	GCACCCGCTGAAYTTAAG	PCR/Sequencing	[61]
1634L	Reverse	ATTCGGCAGGTGAGTTGTTACA	PCR/Sequencing	This study
PCR regime U178/1634L	95°C at 4 min, 2×(94°C at 30 s, 56-54°C at 30 s, (2°C touch down) 72°C at 1 min), 36×(94°C at 30 s, 52°C at 30 s, 72°C at 1 min), 72°C at 10 min			
1200F	Forward	CCCGAAAGATGGTGAACTATGC	PCR/Sequencing	[61]
2450R	Reverse	GCTTTGTTTTAATTAGACAGTCGGA	PCR/Sequencing	
PCR regime 1200F/2450R	95°C at 4 min, 40×(94°C at 30 s, 52°C at 30 s, 72°C at 1 min), 72°C at 10 min			
300F	Forward	CAAGTACCGTGAGGGAAAGTTG	Sequencing	[61]
300R	Reverse	CAACTTTCCCTCACGGTACTTG	Sequencing	[61]
1200R	Reverse	GCATAGTTCACCATCTTTCGG	Sequencing	[61]
UJR2176	Reverse	CGGATCTAATTTGCCGACTTCCCTTA	Sequencing	[62]
1600F	Forward	AGCAGGACGGTGGCCATGGAAG	Sequencing	[61]
**COI Primers**				
LCO1490	Forward	GGTCAACAAATCATAAAGATATTGG	PCR/Sequencing	[63]
HCO2198	Reverse	TAAACTTCAGGGTGACCAAAAAATCA	PCR/Sequencing	[63]
PCR regime LCO1490/HCO2198	95°C at 4 min, 40–45×(94°C at 30 s, 46°C at 30 s, 72°C at 30 s) 72°C at 10 min			

PCR regimes for primer pairs are also given.

In some cases the PCR-product had to be purified with the QIAquick PCR Purification Kit (QIAGEN) according to the manufacturer's instructions.

To remove excess nucleotide fragments EXO and SAP (Fermentas) were mixed in proportions 1∶4 and subsequently 5.5 µl EXOSAP added to all PCR-products. EXOSAP-reactions were run at 37°C for 30 min and 80°C for 15 min. Sequence reactions were made according to the BigDye® Terminator v3.1 Sequencing Standard Kit (Applied Biosystems) following the manufacturer's instructions. An ABI3130XL Automated DNA sequencer (Applied Biosystems, Hitachi) was used to produce chromatograms. Samples which yielded unreadable sequences were cloned using the TOPO TA for Sequencing Cloning Kit (Invitrogen) according to the manufacturer's instructions.

### Alignment and dataset

Contigs were assembled using Staden v 1.6.0 [32]. The consensus sequences were blasted so that contaminations could be discovered. 18S and 28S rDNA sequences were aligned using Muscle [33] with maxiters set to 9999 and maxtrees set to 9999. We used the invertebrate mitochondrial code as a guide in order to only infer gaps between codons. Nucleotide sequences were used in all phylogenetic analyses. However it should be pointed out that when searching for stop codons in our COI sequences using the recent software Translator X [34] the ostensible COI sequences from the two *Oregodasys* species, the two specimens of *Tetranchyroderma thysanophorum*, *Tetranchyroderma esarabdophorum*, *Ptychostomella* sp.1, and *P. tyrrhenica* do not conform to the general invertebrate or any other published mitochondrial genetic code.

Aligned 18S and 28S rDNA were processed so that positions that contained more than 10% gaps were removed using the software Filter (Wallberg unpublished). The combined dataset consisted of 4366 nucleotide characters (1664, 1993 and 654 nucleotide characters for 18S, 28S and COI respectively). For 18S rDNA gene all of the 49 taxa were represented, for 28S 42 taxa were represented and for COI 31 taxa were represented. The dataset was subsequently converted into an interleaved nexus formatted file.

### Phylogenetic analyses and statistics

The combined dataset was analyzed with MrBayes 3.1.2 [35], [36]. Evolutionary models were tested for each of the sequenced genes with FindModel available from the HIV sequence database [37]. The best evolutionary model for each of the sequenced genes were the six parameter general time reversible (GTR) model of nucleotide substitution (nst = 6, rates = invgamma). Two runs with four simultaneous chains were run for 40,000,000 generations. Trees were sampled every 100^th^ generation after a burn in of 10,000,000 generations. The MrBayes analyses were carried out on the Bioportal at Oslo University [38]. For tree drawing Figtree v1.1.2 [39] was used. Consensus trees, as well as filtered groups of trees, were produced with Mesquite [40]. *Xenotrichula intermedia* (Chaetonotida) or species of *Dactylopodola* were used as outgroups.

Moreover a combined analysis of 18S and 28S rDNA was run and compared with the gene tree of COI according to the settings presented above. Gene trees for 18S and 28S rDNA were also obtained by running each individual data set. All analyses were run according to the above settings but for 10,000,000 generations with a burn in of 2,500,000 generations. *Dactylopodola* was used as outgroup for these analyses.

A maximum likelihood analysis using RaxML v7.0.3 [41], [42] with 1000 bootstrap replicates under the GTRCAT approximation was run on the combined dataset to check for congruence with the Bayesian analysis. The optimal tree topology from the ML analysis was subsequently tested against an alternative hypothesis (monophyly of *Tetranchyroderma*) using the approximately unbiased (AU) test [43] using Tree-Puzzle v5.2 [44] and Consel v0.1i [45].

## Results

**Figure 4:**
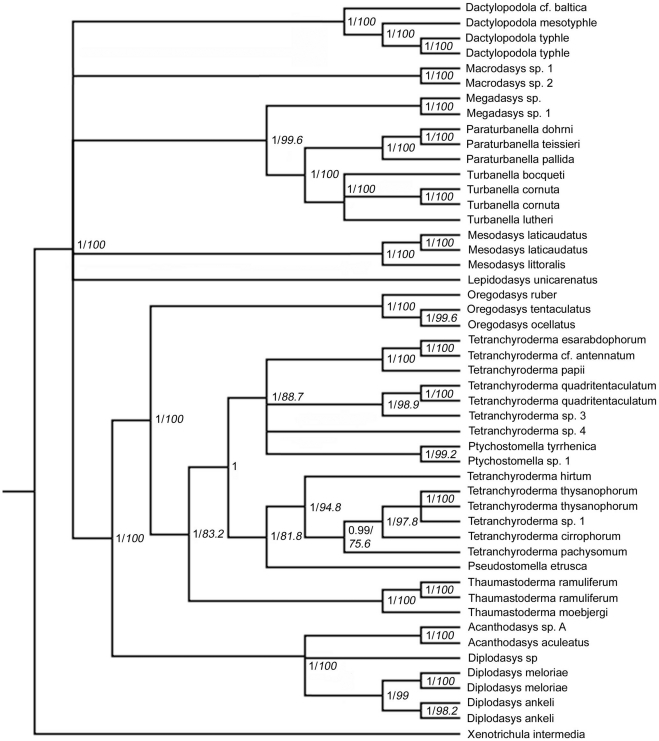
Phylogenetic relationships of Thaumastodermatidae inferred from Bayesian analysis of 18S, 28S rDNA and COI mtDNA. The outgroup is represented by *Xenotrichula intermedia* (Chaetonotida, Xenotrichulidae). Number at nodes represent posterior probabilities (regular) and bootstrap support values (italics). For Bayesian values only nodes with ≥95 are shown (95% majority rule consensus) and for bootstrap support only nodes with ≥75 are shown.

The final alignment of the combined data set consisted of 4366 characters (positions). The phylogenetic analysis with *Xenotrichula intermedia* as outgroup yielded Thaumastodermatidae as strictly monophyletic and both the subfamilies, Thaumastodermatinae and Diplodasyinae, were well supported (Figure 4). Within Diplodasyinae, *Diplodasys* appear non-monophyletic, due to the uncertain position of a single species of *Diplodasys* out of five terminals. Within Thaumastodermatinae, the genus *Oregodasys*, here represented by the three species, *O. ocellatus*, *O. ruber* and *O. tentaculatus*, was monophyletic and turned out as basal to all other taxa within the group. The monophyly of *Thaumastoderma* represented by three specimens from two species, was highly supported as well. The genus appears here as the sister taxon of the remaining taxa. The more densely sampled *Tetranchyroderma*, was non-monophyletic and formed two well supported clades where one assemblage of species appeared as the sister-group to *Pseudostomella* and the other clustered with *Ptychostomella*. The first clade contains *Tetranchyroderma cirrophorum*, *T. hirtum*, *T. pachysomum*, *T. thysanophorum* and *Tetranchyroderma* sp. 1 as a sister group to *Pseudostomella etrusca*. The second clade has *Ptychstomella* sp. 1 and *P. tyrrhenica* nested within a group containing *T.* cf. *antennatum*, *T. esarabdophorum*, *T. papii*, *T. quadritentaculatum*, *Tetranchyroderma* sp. 3 and *Tetranchyroderma* sp. 4.

**Figure 5:**
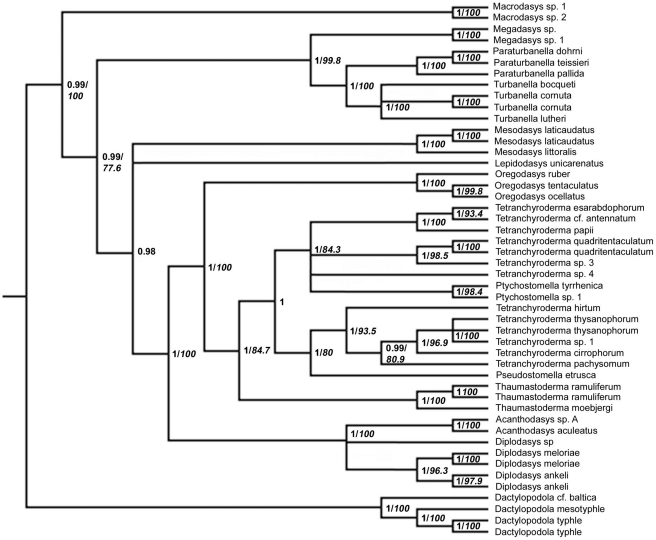
Phylogenetic relationships of Thaumastodermatidae inferred from Bayesian analysis of 18S, 28S rDNA and COI mtDNA. The outgroup is represented by *Dactylopodola* cf. *baltica*, *D. mesotyphle* and *D. typhle* (Macrodasyida, Dactylopodolidae). Number at nodes represent posterior probabilities (regular) and bootstrap support values (italics). For Bayesian values only nodes with ≥95 are shown (95% majority rule consensus) and for bootstrap support only nodes with ≥75 are shown.

The phylogenetic analysis of the combined data set with *Dactylopodola* species as outgroup was in general accordance with the analyses where *Xenotrichula intermedia* served as outgroup. The non-monophyly of *Tetranchyroderma* is retained in this analysis as well and the within-family groupings are the same as in the *Xenotrichula intermedia* outgroup-bearing phylogeny (Figure 5). In general, statistical support at nodes are higher in this analysis, whereas slight differences regard alliances among non-Thaumastodermatidae taxa.

The combined data set of the nuclear genes contained 3663 positions; the results were in general accordance with the species tree based on the combined nuclear and mitochondrial data set. The only notable exception was that the two specimens of *T. quadritentaculatum* were in a sister group relation to *Thaumastoderma* (Figure S1).

Analyses of individual gene trees indicated that the nuclear genes (18S rDNA and 28S rDNA) have little, if any, conflicts. The 18S gene tree is better resolved than the 28S gene tree. In both trees Thaumastodermatidae emerge as a well supported group and within this clade the subfamilies Diplodasyinae and Thaumastodermatinae were also well supported. Within Thaumastodermatinae, *Oregodasys* has a basal position in both gene trees. The large genus *Tetranchyroderma* is non-monophyletic in both trees (Figures S2, S3).

In the 18S gene tree, *P. etrusca* is a sister group to all other *Tetranchyroderma* except *T. quadritentaculatum*. *Ptychostomella* is nested within *Tetranchyroderma* in a clade together with *T.* cf. *antennatum*, *T. esarabdophorum*, *T. papii*, *T.* sp. 3 and *T.* sp. 4.

In the 28S gene tree, *P. etrusca* is nested within a poorly resolved clade together with *Tetranchyroderma* and *Thaumastoderma*. *Ptychostomella* is nested in a poorly resolved clade with the same species as in the 18S gene tree (Figures S2, S3).

The basal parts of the COI gene tree are very poorly resolved. *Tetranchyroderma* is non-monophyletic in a clade together with *Oregodasys*, *Ptychostomella* and *Pseudostomella*. Moreover, the COI phylogeny is in conflict with the nuclear gene trees for example regarding the position of *Oregodasys*, which is not basal in Thaumastodermatidae but nested within *Tetranchyroderma* (Figure S4). Nevertheless, including COI in the concatenated phylogenetic analysis increased support values for several clades compared to the 18S and 28S gene trees.

With regard to the other taxa, two families are here represented by more than one genus: Turbanellidae, which appeared to be monophyletic, and Cephalodasyidae, which was resolved as polyphyletic, with *Megadasys* shown in alliance with Turbanellidae, and *Mesodasys* standing alone. All the other single genera formed a polytomy (Figure 5). Our analyses failed to find the sister group of Thaumastodermatidae among the species/taxa examined here.

The ML phylogeny was congruent with the Bayesian phylogeny. Bootstrap support values were high for Thaumastodermatidae as well as for Thaumastodermatinae and Diplodasyinae. The basal position of *Oregodasys* within Thaumastodermatinae was strongly supported and so was the sister group relationship of Thaumastoderma with the remaining taxa. Likewise *Tetranchyroderma* was non-monophyletic with the same groupings as in the Bayesian analysis. The approximately unbiased test based on the ML analysis rejected a constraint tree where *Tetranchyroderma* was kept monophyletic (p<0.01) compared to the best scoring ML-tree (Table 4).

**Table 4:** Output from Consel.

Tree	au
1 (Constrained monophyly of *Tetranchyroderma*)	0.008
2 (Optimal ML tree)	0.992

Tree 1 has the constraint topology where *Tetranchyroderma* is monophyletic.

## Discussion

In analysis of the concatenated dataset two different outgroups were used: (i) *Xenotrichula intermedia* (Chaetonotida, Xenotrichulidae) and (ii) *Dactylopodola* cf. baltica, *D. mesotyphle* and *D. typhle* (Macrodasyida, Dactylopodolidae). Use of these different outgroups did not alter the general topology of the tree, although for the concatenated tree resolution was better and support values were a little higher with *Dactylopodola* as outgroup. Because of this, *Dactylopodola* was also used as outgroup for the individual gene trees.

The combined phylogeny of 18S, 28S and COI gives the same general results as the combined phylogeny of the two nuclear genes as well as the gene trees of 18S rDNA and 28S rDNA. Also, the maximum likelihood analysis is congruent with these results, so was the result of a parallel analysis conducted using a Maxium Parsimony method (Figure S5).

All of this suggest that the phylogenetic scenario recovered by the topology in the concatenated analysis (Figure 4) is extremely robust (i.e. very likely).

The hypothesis that Thaumastodermatidae - the largest and one of the most morphologically diverse macrodasyidan families - is monophyletic gains strong support in our study. Rieger & Rieger [46] also confirm monophyly of the family based on cuticular ultrastructure, as does Ruppert [23] based on the structure/function of the reproductive organs.

In this framework, it seems interesting to discuss the in-group evolutionary hypotheses put forward by previous authors in the light of our results. For instance, considering the features of the cuticular covering together with the traits of the reproductive apparatus, the two subfamilies Diplodasyinae and Thaumastodermatinae gain high support as monophyletic groups, having a sister group relationship [e.g. 18], [30]. Moreover, morphology seems not to leave doubts about the monophyly of the currently recognized genera and so far taxonomists have not raised concerns in this regard.

In our analyses, the two subfamilies are resolved as monophyletic with high statistical support and their sister group relationship is corroborated.

Regarding the monophyly of the genera, within Diplodasyinae both genera in the group were represented in our analysis containing seven terminals in total: two putative species for *Acanthodasys* and three species and 5 specimens for *Diplodasys*. In our study *Acanthodasys* appears monophyletic while *Diplodasys* is unresolved due to the uncertain position of *Diplodasys* sp., a new species collected in Kuwait (Figs. 1, 2). Considering that the other four *Diplodasys* terminals cluster together with a high statistical support and knowing that only minor taxonomic characters (e.g. number of adhesive tubes, presence of cephalic sensorial organs etc) distinguishes the Kuwaiti specimens from other known *Diplodasys* species, there seem to be realistic reasons to consider *Diplodasys* as a monophyletic taxon.

According to Hochberg & Litvaitis [18], [30], the subfamily Thaumastodermatinae, which includes five extant genera, may be defined by a single morphological autapomorphy: the loss of the left testis. However, other characteristics may distinguish Thaumastodermatinae from Diplodasyinae e.g., by the presence of caudal and frontal organs in posterior trunk region and adjacent to each other, and an extensively modified cuticular covering. Differences in these traits have been used to infer in-group phylogeny; results of these studies however have not always been congruent with each other [e.g. [18] vs [19]]. One exception is the sub-clade that includes *Pseudostomella* as the sister group of *Tetranchyroderma* plus *Thaumastoderma* that has consistently been recovered in the cladistic analyses, and at least twice with a reasonably high statistical support [e.g. 18], [30]. Species of this grouping are characterized by elaboration of cuticular spines into 3-, 4-, and 5-pronged hooks, which has been presumed to be derived from an ancestral single-spined, bowl-shaped scale retained in Diplodasyinae [46].

Some evidence [30] suggests that the sister-group of this assemblage could be a clade formed by *Oregodasys* (formerly known as *Platydasys*) and *Ptychostomella* whose representatives are typically characterized by a non-armored cuticle, a condition presumably derived through loss of the bowl-shaped scales [46].

In this scenario, the central spine of the five-pronged spines ( = pentancres) found in some Thaumastodermatinae (i.e. *Pseudostomella* and *Tetranchyroderma*) would be homologous to the central spine of the bowl-shaped scale in *Diplodasys* and *Acanthodasys*, while the external four spines are probably local specializations of the bowl-shaped scale found in Diplodasyinae. Within this framework, the four- pronged scales found in other species of *Pseudostomella*, *Tetranchyroderma* and in all species of *Thaumastoderma* developed from five-pronged scales by reduction of the central spine [46], [ see also 6]. Perhaps the same process that led to the reduction of number of spine could be invoked to explain the existence of the less common three pronged scales (triancres) present in other ancrous species of Thaumastodermatinae [47] not considered by Rieger & Rieger [46].

In our study, the larger subfamily Thaumastodermatinae is basally well resolved. *Oregodasys* formed the sister group of all other Thaumastodermatinae. A basal position of *Oregodasys* agrees with the hypothesis of Ruppert [23] who studied the reproductive organs within Thaumastodermatidae and indicated the caudal organ of *Oregodasys* to be both male and female in function, a condition he speculated to be close to the ancestral state of Thaumastodermatidae. Considering the relevant implication of this hypothesis, it appears quite surprising that the author did not invigorate this idea in his later master work [6]. In our opinion the structure and function of the accessory reproductive organs of *Oregodasys* is not fully understood, so it appears unwise to take for granted a basal position of this taxon on the basis of unconfirmed studies.

Our well-supported results encourage new studies of the reproductive apparatus of these bulky thaumastodermatids to test the findings and hypothesis of Ruppert [23]. Additional information could help, in an evolutionary perspective, to understand how reproduction is achieved in Thaumastodermatidae. New information would benefit also future morphology-based phylogenetic analyses, the result of which, due to different characters and character state scoring, have so far yielded contrasting results with regard to the position of *Oregodasys*: e.g. a basal position in Hochberg and Litvaitis [18], more derived line in Hochberg & Litvaitis [30] and in Kieneke *et al* [19].

Our study also suggests that future phylogenetic studies based on morphology should consider the absence of scales/spines in *Oregodasys* and *Ptychostomella* not to be a homologous trait and consequently score this characteristic accordantly; this hypothesis is further corroborated by the differences that exist between the cuticular covering of the two taxa at both light microscopy and ultrastructural levels [e.g.], [ 48,49].

The basal postition of *Oregodasys* along the Thaumastodermatinae branch may raise a question about the ancestral state of the cuticle in Thaumastodermatidae: armoured or smooth? Several evidences point to the first as the ancestral state, among others: (i) scales and spines are present in members of most genera of the family, including *Acanthodasys* and *Diplodasys* that possess other ancestral characteristics e.g., paired testes; (ii) abundance of epidermal glandulocytes in *Oregodasys* [50]. Ruppert [6] hypothesized a repugnatorial function for the glandulacytes, so vicariating the protective function of scales and spines by producing toxic and/or repellent material as defence against predation. This hypothesis is supported by the subsequent discovery of *Tetranchyroderma* species that are characterized by a reduction of the cuticular armature (bikini-trix, see below) and an abnormal high number epidermal glands.

Our study found a well-supported monophyletic *Thaumastoderma* to be the second-most basal taxon along the Thaumastodermatinae phylogenetic branch, an evolutionary hypothesis never formulated before. Our results shed light on the origin and evolution of the one of the most striking morphological features of these gastrotrichs, i.e., the ancestral shape of the anchored spines, whose appearance is responsible for the taxon name, *Thaumastoderma* (Latin: = miraculous skin).

The position of *Thaumastoderma* as basal to the reminder of the ancre-bearing taxa implies that their ancestor probably had a tetrancrous covering (i.e. tetrancres as primary states) with pentancres evolving secondarily in the common ancestor of *Tetranchyroderma* and *Pseudostomella*. In this scenario the central prong that characterizes the pentancres is a secondary acquisition and therefore not homologous with the central spine of species of Diplodasyinae as previously hypothesized (see above). Furthermore, while the central spine, and hence pentancre, may be a synapomorphy of *Tetranchyroderma* and *Pseudostomella*, its seemingly distribution throughout both genera may also imply independent evolution in both taxa.

Our study also indicates that the speciose genus *Tetranchyroderma* is non-monophyletic, a result strongly supported in both Bayesian and ML analysis; of the 600000 trees that were sampled during the analyses none contained a monophyletic *Tetranchyroderma*. The alternative hypothesis (monophyly of *Tetranchyroderma*) was rejected by the approximately unbiased test (p<0.01). It appears from our results that the genus should be split into at least two groups, of which one should be affiliated to *Pseudostomella* and the other should encompass also *Ptychostomella* (Figure 1).

A sister group relationship between *Pseudostomella* and *Tetranchyroderma* has been shown in several studies [e.g. 11], [18], [30]. However, it is difficult to compare our findings with the results from previous phylogenetic analyses due to differences in methodologies and taxonomic sampling. For instance the studies based on morphology [18], [30] have considered the two taxa as single terminals while only two *Tetranchyroderma* species were involved in the molecular study of Todaro *et al.* [11]. All considered, it is realistic to believe that the past results may have been biased by the poor taxonomic sampling; the poorness regards both the number but also the type of the species involved in the past studies. This statement finds support considering that there is a conflict between our study and Todaro *et al.* [11] regarding the relative position of *Pseudostomella* and *T. papii*, which are mutually exclusive in the two studies. It should be highlighted that the position of *Pseudostomella* was unstable in additional analyses performed with a subset of the taxa in our dataset (not shown).

In the species-based cladistic analysis of Gastrotricha by Kieneke *et al.* [19] that included two *Pseudostomella* and three *Tetranchyroderma* species, a monophyletic *Pseudostomella* was found basal to most thaumastodermatids, except *Acanthodasys* and *Thaumastoderma* while *Tetranchyroderma* appeared paraphyletic; again bootstrap values at nodes was in all cases very low, leaving little confidence on these results.

In our opinion, a sister-group relationship between *Pseudostomella* and at least some *Tetranchyroderma* species is most likely, based for example on the potential to evolve five- and three-pronged scales, starting from the ancestral tetrancres. A monophyletic *Pseudostomella* is also credible, based on the impressive autoapomorphy constituted by the oral palps, a characteristic without equivalent in any other gastrotrich taxa (Figure 3).

Our most surprising result is the splitting of *Tetranchyroderma* into two clades. This is because we cannot think of any morphological synapomorphies of the two groups that can be used as diagnostic features, except perhaps the fact that species allied with *Pseudostomella* lack cephalic tentacles, whereas cephalic tentacles, rod- and/or knob-like are present in the species that cluster with *Ptychostomella*. It is also possible that the importance of the phylogenetic signal of other characters (e.g. arrangement of the anterior adhesive tubes, structure of the caudal pedicles, shape and structure of the fronto-caudal organ complex etc.) may have thus far been overlooked.

In comparison, the relationship of certain *Tetranchyroderma* species with *Ptychostomella* may be simpler to explain. The monophyly of *Ptychostomella* is supported by our analysis; however, morphologically the group is distinguished on a negative character i.e., the absence of scales and/or spines (Figure 3). A possible explanation for the rise of the *Ptychostomella* lineage would be that the group shares a common ancestor with a subset of the *Tetranchyroderma* species characterized by a tendency toward reduction and loss of the cuticular hooks. This hypothesis receives support based on the overall resemblance of their external and internal anatomies to small species of *Tetranchyroderma* than to any other thaumastodermatids. Moreover, the reduction of the cuticular covering is a phenomenon all but infrequent in *Tetranchyroderma* as testified by the presence of the so-called “bikini-trix” [41], [52] a complex of 5–6 species characterised by incomplete cuticular covering, which in *T. hypopsilacrum*, for instance, may be restricted to some epaulets in the pharyngeal region [cf 25]. If corroborated by further studies this hypothesis would make *Tetranchyroderma* paraphyletic, creating even more conflicts with the current classification.

To summarize, the concatenated phylogeny is congruent with other studies dealing with in-group relationships in Macrodasyida. Thaumastodermatidae has been found to be monophyletic based on morphology by Hochberg & Litvaitis [18], [30] and Kieneke *et al.* [19]. Ruppert [23] states that the possibility of polyphyly is remote and gives the following apomorphies for a monophyletic Thaumastodermatidae: (i) complex cuticle, (ii) structure of the pharynx, (iii) lack of circular muscles in the lateral body regions, (iv) internal connection of vas deferens or vas deferentia to the caudal organ, and (v) multiciliated epidermal cells. Molecular studies are concordant with morphology and the group has been found monophyletic based on 18S rDNA by Todaro *et al.* [11] and Petrov *et al.* [21]. The only other investigation where some of the internal relationships within the family were studied is Todaro *et al.* [11], which found high support for the monophyly of Diplodasyinae and Thaumastodermatinae. Moreover the same study also found a sister group relationship between *Pseudostomella etrusca* and two species of *Tetranchyroderma*. A similar sister group relation is also presented in this study where approximately half of the sampled *Tetranchyroderma* form a sister group relation to *P. etrusca*.

Traditionally the number of prongs on scales of *Tetranchyroderma* has been used to discriminate between species and subgroups within the genus. In our analysis taxa with different number of prongs clusters together. *T. antennatum*, *T. esarabdophorum*, *T. hirtum*, *T. papii*, *T. quadritentaculatum*, *T.* sp. 1, *T.* sp. 4 and T. *thysanophorum* all have five pronged scales [51], [52]–[56, M.A. Todaro unpublished] while *T. cirrophorum*, *T. pachysomum*, *T.* sp. 3 have four pronged scales [51], [57, M. A. Todaro unpublished]. Hence, the number of prongs on scales is not a good morphological character to use for determining relationships within *Tetranchyroderma*, although it is extremely useful in dichotomous keys [cf 25].

Regarding the relationships of other families exclusive of Thaumastodermatidae, a few comments can be made. Turbanellidae are monophyletic and congruent with traditional classification within Macrodasyida. The non-monophyly of the Lepidodasyidae sensu Remane [58] has been known for some time [23]; our results support the recent separation of *Lepidodasys* from other genera previously affiliated with the family [7]; however, it seems that the revisional work is not finished as the position of *Megadasys* and *Mesodasys* in our tree suggests that also the new erected family (i.e. Cephalodasyidae) may be non-monophyletic.

Finally, the ostensible COI sequences from the two *Oregodasys* species, the two specimens of *Tetranchyroderma thysanophorum*, *Ptychostomella* sp1 and *P. tyrrhenica* do not conform to the general invertebrate or any other published mitochondrial genetic code. We re-examined all COI chromatograms and re-amplified and sequenced the six specimens with identical results. The sequences we have obtained (Accession numbers: JF432020, JF432021, JF432022, JF432027, JF432025, JF432033, JF432034) may be mitochondrially derived nuclear genes (numts), or there may have been one or more modifications of the mitochondrial genetic code within Gastrotricha.

## Supporting Information

Figure S1 **Phylogenetic relationships of Thaumastodermatidae inferred from Bayesian analysis of 18S rDNA and 28S rDNA (95% consensus tree).** The outgroup is represented by members of *Dactylopodola*. Number at nodes represent posterior probabilities.
(TIF)

Figure S2 **Phylogenetic relationships of Thaumastodermatidae inferred from Bayesian analysis of 18S rDNA (95% consensus tree).** The outgroup is represented by members of *Dactylopodola*. Number at nodes represent posterior probabilities.
(TIF)

Figure S3 **Phylogenetic relationships of Thaumastodermatidae inferred from Bayesian analysis of 28S rDNA (95% consensus tree).** The outgroup is represented by members of *Dactylopodola*. Number at nodes represent posterior probabilities.
(TIF)

Figure S4 **Phylogenetic relationships of Thaumastodermatidae inferred from Bayesian analysis of COI mtDNA (95% consensus tree).** The outgroup is represented by *Xenotrichula intermedia*. Number at nodes represent posterior probabilities.
(TIF)

Figure S5 **Phylogenetic relationships of Thaumastodermatidae inferred from Maximum Parsimony analysis of 18S, 28S rDNA and COI mtDNA.** The outgroup is represented by *Xenotrichula intermedia* (Chaetonotida, Xenotrichulidae). Tree # 1 out of 3 most parsimonious trees (length = 12473) is shown. Number at nodes represent bootstrap support values (1000 replicates). The MP analysis was conducted with MEGA 4 using the default settings.
(TIF)
