# Supplementary figures and images for: Phylogeny of Thaumastodermatidae (Gastrotricha: Macrodasyida) Inferred from Nuclear and Mitochondrial Sequence Data
Source: PLoS One. 2011 Mar 24;6(3):e17892. doi: 10.1371/journal.pone.0017892 (PMC3063787; doi:10.1371/journal.pone.0017892)

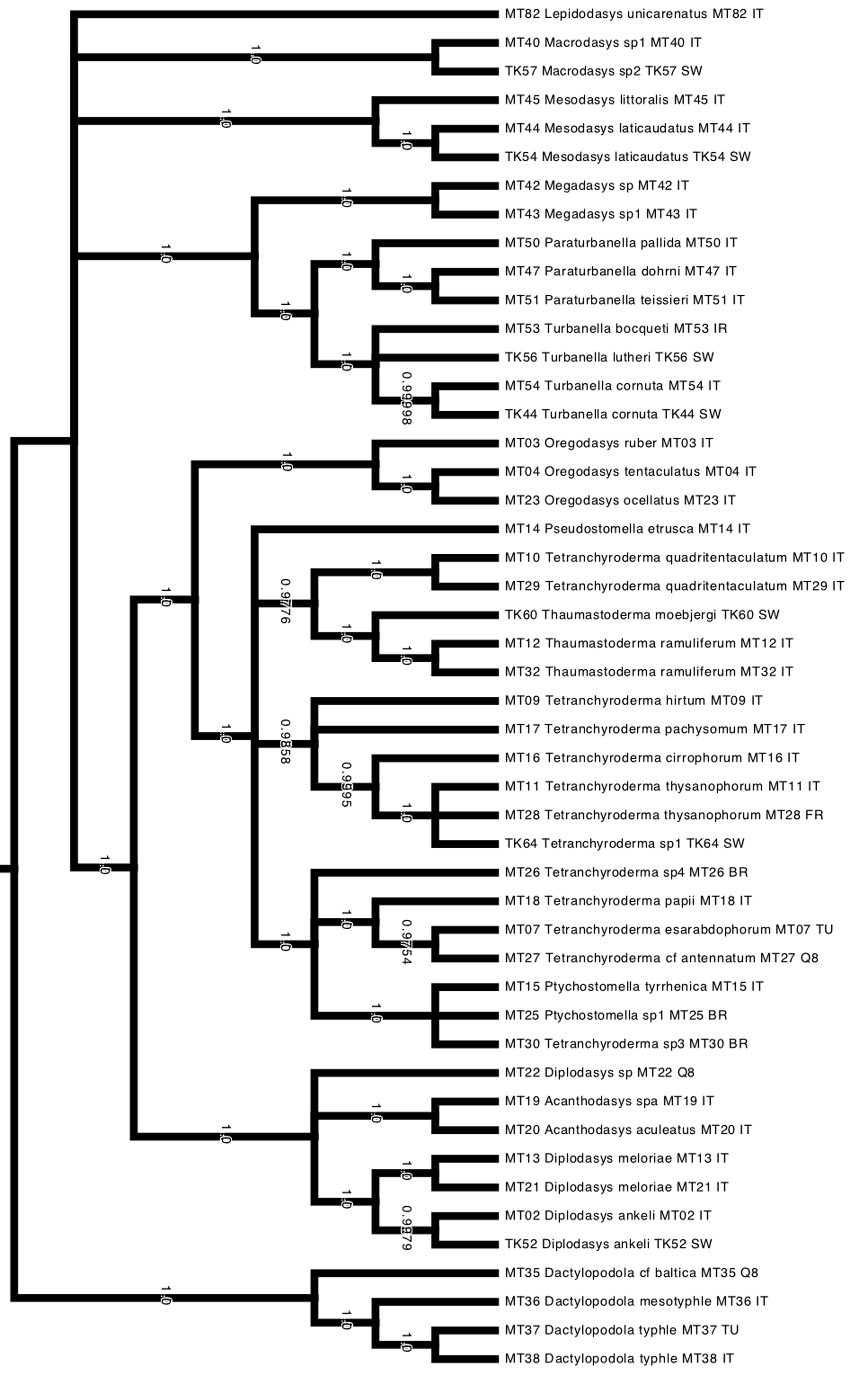

Supplement: Figure S1 — Phylogenetic relationships of Thaumastodermatidae inferred from Bayesian analysis of 18S rDNA and 28S rDNA (95% consensus tree). The outgroup is represented by members of Dactylopodola. Number at nodes represent posterior probabilities. (TIF) [file pone.0017892.s001.tif]

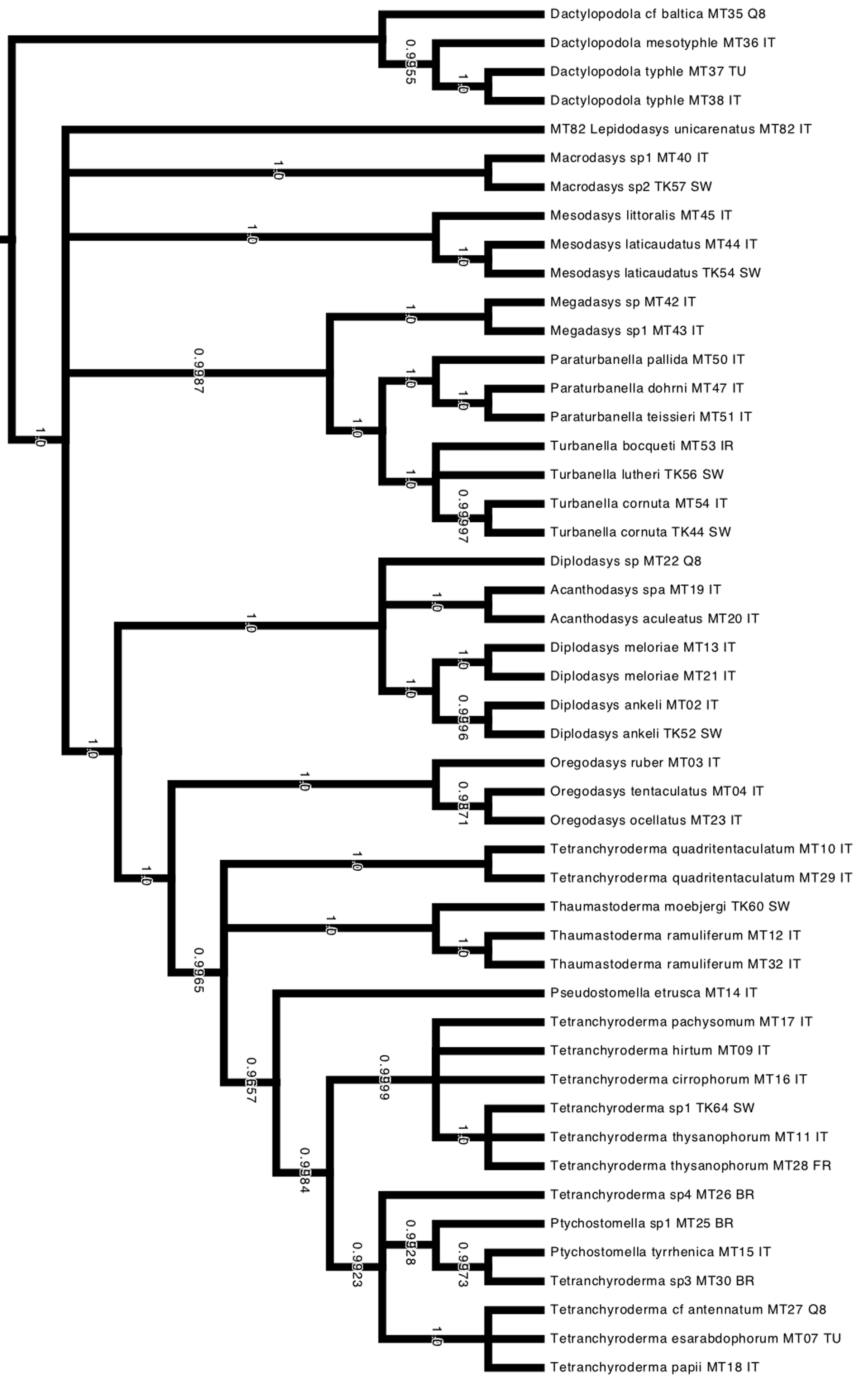

Supplement: Figure S2 — Phylogenetic relationships of Thaumastodermatidae inferred from Bayesian analysis of 18S rDNA (95% consensus tree). The outgroup is represented by members of Dactylopodola. Number at nodes represent posterior probabilities. (TIF) [file pone.0017892.s002.tif]

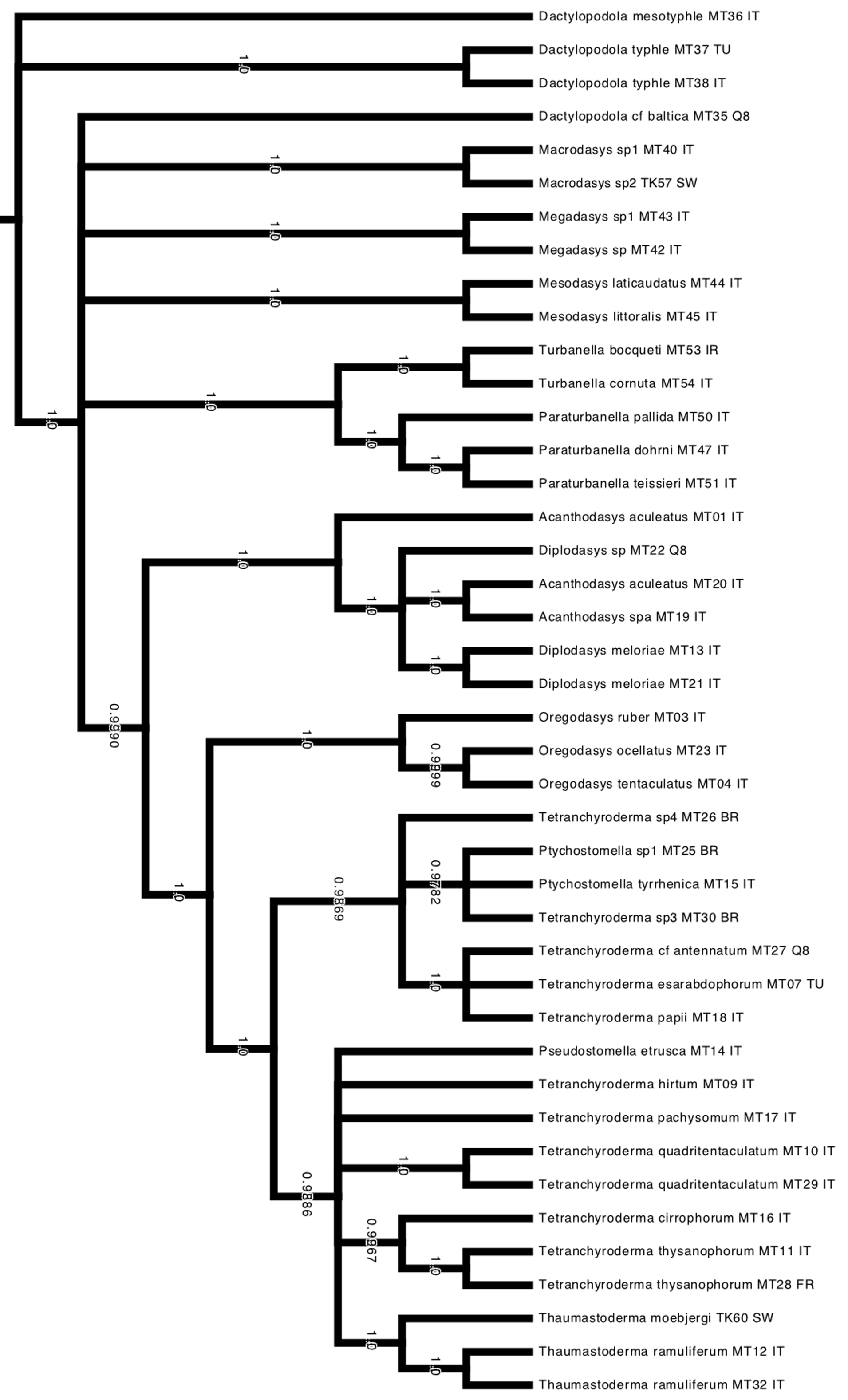

Supplement: Figure S3 — Phylogenetic relationships of Thaumastodermatidae inferred from Bayesian analysis of 28S rDNA (95% consensus tree). The outgroup is represented by members of Dactylopodola. Number at nodes represent posterior probabilities. (TIF) [file pone.0017892.s003.tif]

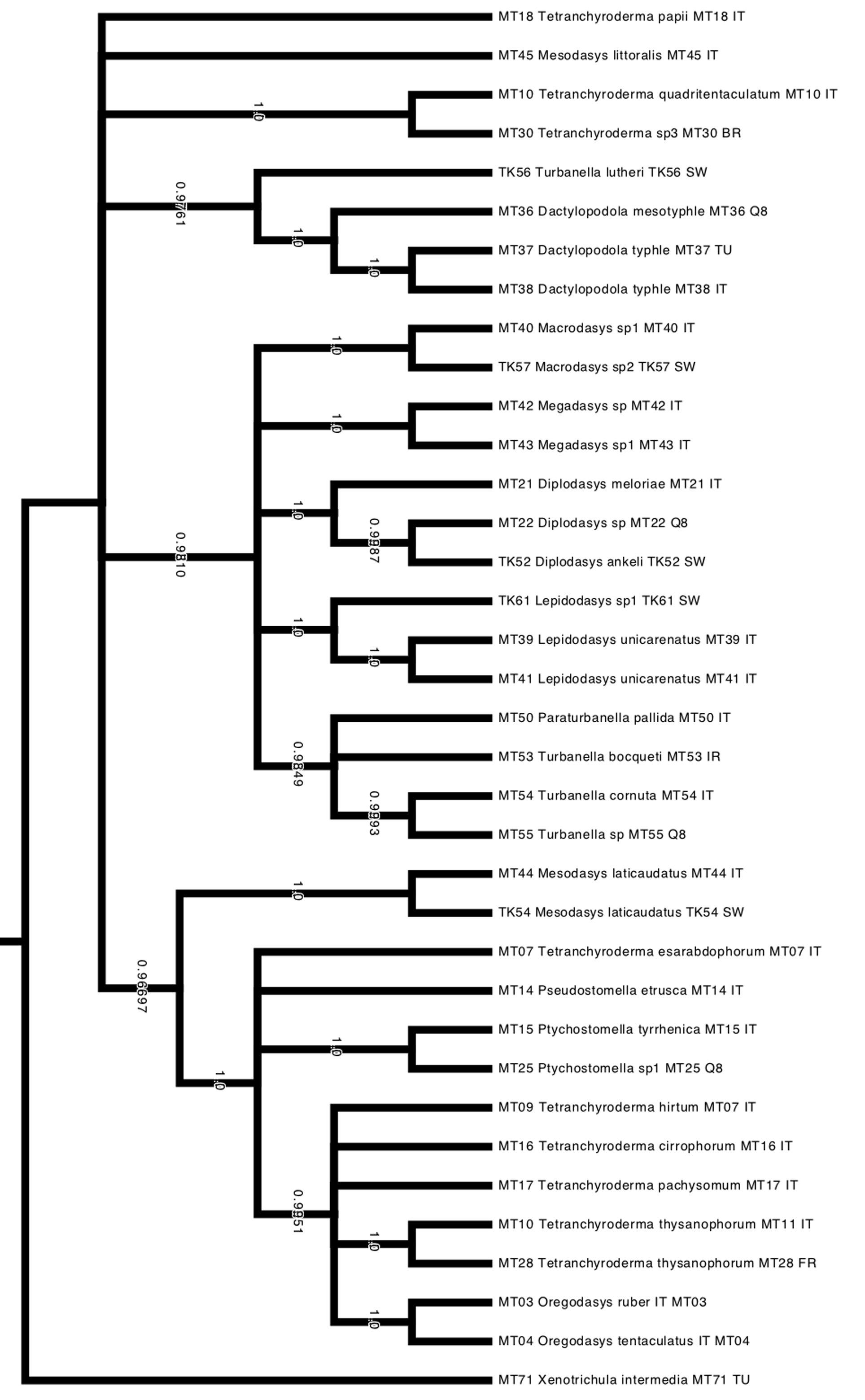

Supplement: Figure S4 — Phylogenetic relationships of Thaumastodermatidae inferred from Bayesian analysis of COI mtDNA (95% consensus tree). The outgroup is represented by Xenotrichula intermedia. Number at nodes represent posterior probabilities. (TIF) [file pone.0017892.s004.tif]

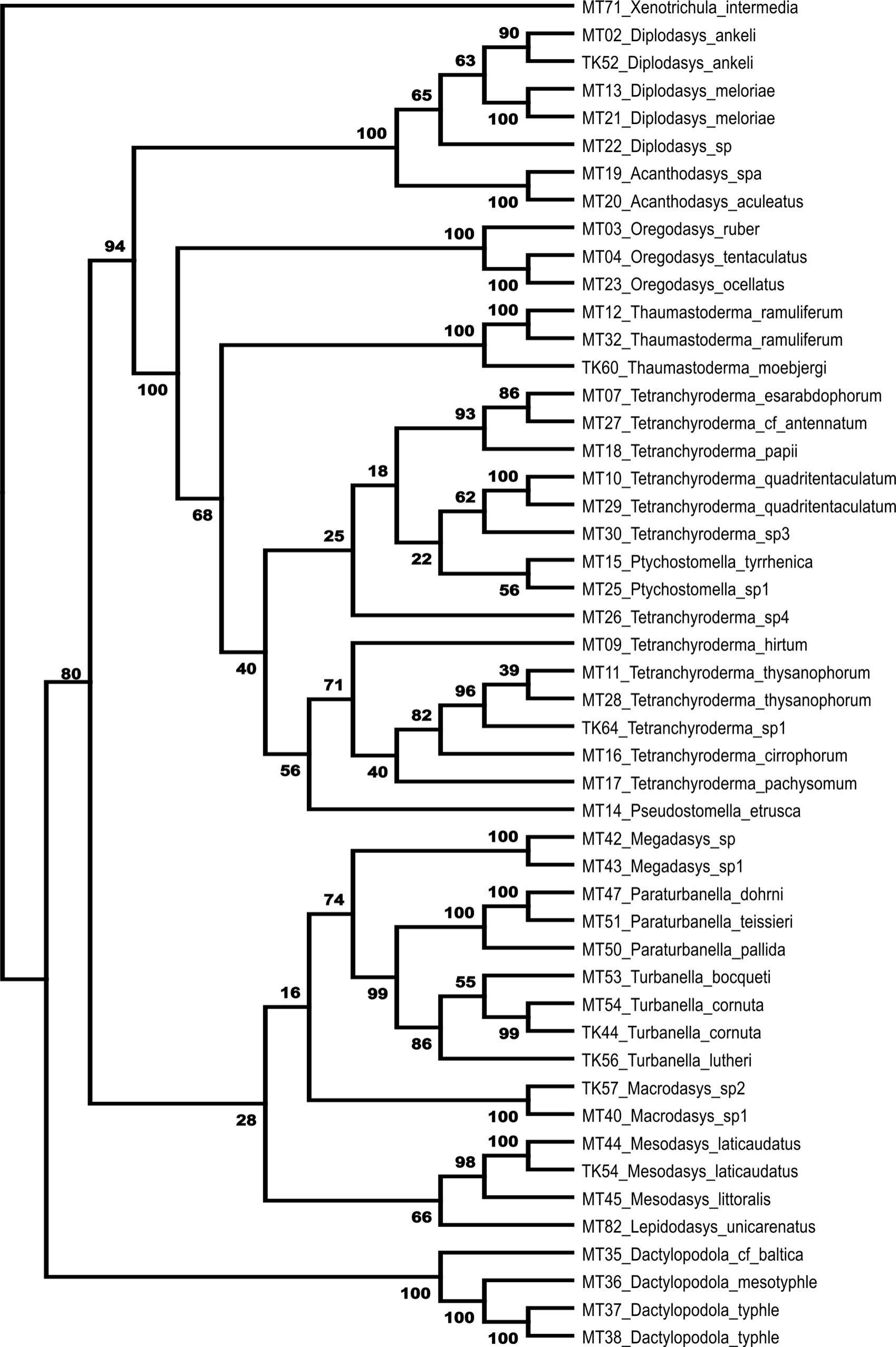

Supplement: Figure S5 — Phylogenetic relationships of Thaumastodermatidae inferred from Maximum Parsimony analysis of 18S, 28S rDNA and COI mtDNA. The outgroup is represented by Xenotrichula intermedia (Chaetonotida, Xenotrichulidae). Tree # 1 out of 3 most parsimonious trees (length = 12473) is shown. Number at nodes represent bootstrap support values (1000 replicates). The MP analysis was conducted with MEGA 4 using the default settings. (TIF) [file pone.0017892.s005.tif]
